# Proposals in France for Renumerating Dr. Jenner

**Published:** 1807-10

**Authors:** James Clarke

**Affiliations:** Nottingham


### To Dr. BATTY.

SIR,

I AM induced to present you for insertion in the next number of your useful Journal the following translation, expressive of the public opinion in France, on the merit due to the benevolent Jenner, for two reasons.

1. The additional grant of parliament to the discoverer, has been thought by some superfluous and profuse.

2. Others more liberal argue, that if vaccination be that blessing its supporters assert, which they are willing to believe, from the clear and concise report the College of Physicians have laid before parliament, the grant cannot be considered in any other light than a mere reimbursement.

I am, Sir,

Yours, respectfully,

JAMES CLARKE, M. D.

*Nottingham, Sept.* 9, 1807.

---

### *A Project of Remunerating* Dr. JENNER, *proposed by* Dr. VALENTINE, *of Marseilles.**

Ten years have elapsed since Dr. Jenner proved the vaccine inoculation a certain preventive against small-pox.

---

* See Journal General de Medicine for March 1807.

It is more than thirty years since he made his first experiments on the nature of cow-pox. Nine years since he published this valuable discovery, and nearly seven years that it has been introduced into France. The practice is at present extended nearly over the whole globe. Many millions have experienced its blessings and every day is marked with the multiplied and uniform success.

Two years were scarcely elapsed when the question appeared completely resolved. There cannot now be any doubt of the possibility of annihilating, by vaccination, the most horrible and most fatal of all diseases.

Have we not always considered this discovery inestimable, and above all comparison, the most important which has been made for the happiness of mankind? Are we not ourselves eager to practice it, and to extend its advantages by every means in our power? We instantly abandoned the inoculation of small-pox, as soon as we were assured that the inoculation of the vaccine was a certain preventative of that disease. Devoted from our profession to combat against the maladies of human nature, we have consolation in not having neglected any thing that might tend to the general good. Truth, often slow in her progress, has conquered by our mutual efforts with an astonishing rapidity.

What acknowledgments do we not owe to the author of this new practice! Let all the world bless him. Every country, every city should offer him a civic crown, and each individual express his gratitude. What man ever performed so much for society! none; no recompense, no dignity, can sufficiently repay such unexampled benefit. The noble and generous manner with which Jenner communicated his discovery, the desire he had to make known the result of his experience, are above all eulogy. As he produced an extraordinary revolution in this important part of medicine for the good of his fellow-creatures, by a practice as simple, as singular, and remarkable; so he did not value to perfect his plan, neither the time, trouble, nor the expence which is occasioned by an immense correspondence.

The medical faculty of France have not been backward to acknowledge him the *Benefactor of Mankind;* and he is thus declared by public opinion. The central committee of vaccine established in Paris, under the auspices of government, and to which France is indebted for the first essays on the new inoculation, as well as the greater part of the happy consequences to the zeal of its members, says, says, in the Report published in 1803, "The committee cannot finish this report of its present progress, without paying, in the name of the subscribers, a just tribute of acknowledgment to Dr. Jenner, the illustrious author of the discovery, who hereafter will be ranked among those who have most honoured science to benefit mankind."

The reward which the English parliament voted to Jenner in 1802, although accompanied with language extremely honourable, is very far below the incalculable advantages resulting from his discovery. The English nation, under Queen Anne, overwhelmed the Duke of Marlborough with honours. That for his feats in war, she should present him with the estate of Woodstock, where she vauntingly built the magnificent Blenheim, and on an elevation in the park, a superb monument, the base of which, covered with inscriptions, attest his warlike exploits; and the summit supports the statue of the general, is not astonishing. But it is very remarkable that this nation, since 1802, has not done any thing more for Jenner. We have seen only in 1805, that the Lord-Mayor and the Corporation of London, have shewn him proof of the public gratitude in presenting him with the freedom of the city in a gold box set with diamonds, and scientific emblems of the salutary discovery of vaccination.*

Many inhabitants of the East Indies, principally in Bengal and Madras, being struck at the small grant of the British parliament, opened a subscription, the produce of which will be presented to Jenner, who has procured to them the means of extirpating from their country the most destructive plague.

Jenner is become a native of all nations. Like Hippocrates, he belongs to all countries His name will exist to eternity, even in the most changeable times. It is the present generation that owe him a suitable remuneration. May she prove worthy of the most enlightened period of the world! May the French nation, which knows how to appreciate great acts, no longer delay.†

* Since Dr. Valentin addressed to the public the above very just reproach, Parliament having received sufficient evidence, drawn up in the most able manner by the College of Physicians of the increasing utility of, and dependance on vaccination, has thought it proper to make a further grant to Dr. Jenner of 20,000l. T.

† Edward Jenner was born at Berkley, in Gloucestershire, the 17th of May 1749.

After these remarks, I would propose to all the Societies formed in the French empire for the advancement and improvement of Medicine:

1st. To open with the consent, and under the protection of government, a subscription in favour of Dr. Jenner.

2d. The committee of the central Society of Vaccine and the Societies of Medicine in the metropolis, should have exclusively the power of determining the nature of the remnueration to be presented to this great man.

3d. These societies should appoint some of their members to present them with a plan to this effect, and to obtain from his excellency the Minister of the interior, the permission to invite the Societies of Medicine of the departments to contribute to the gift by voluntary subscription.

4th. All the Literary Societies, particularly those which profess medicine, all the members of the Committee of Vaccination, should be at liberty to contribute to it.

5th. At a time fixed for the close of the subscriptions, the commission formed by the Societies of Paris should name the deputies to go to England, as soon as circumstances and the government may permit, to express our respect and our gratitude to Dr. Jenner.

6th. The same commission should determine also the time and place where it may be convenient to erect a statue of him.

7th. It is presumed, that the Societies of Medicine will themselves place a bust of Jenner by the side of that of Hippocrates and Napoleon I.

*Marseilles, Feb.* 6, 1807.

